# Evaluating the biomechanical performance of Ti6Al4V volar plates in patients with distal radius fractures

**DOI:** 10.3389/fbioe.2023.1141790

**Published:** 2023-02-27

**Authors:** Hua Li, Daofeng Wang, Wupeng Zhang, Cheng Xu, Dou Xiong, Jiantao Li, Licheng Zhang, Peifu Tang

**Affiliations:** ^1^ Senior Department of Orthopedics, The Fourth Medical Center of Chinese PLA General Hospital, Beijing, China; ^2^ National Clinical Research Center for Orthopedics, Sports Medicine and Rehabilitation, Beijing, China; ^3^ School of Medicine, Nankai University, Tianjin, China

**Keywords:** biomechanical performance, Ti6Al4V, volar plates, distal radius fractures, simulation analysis

## Abstract

**Purpose:** This study aimed to investigate the biomechanical performance of three Ti6Al4V volar plates with the latest designs using a finite element model.

**Methods:** An AO type 23-A3 distal radius fracture and the models of T plate (2.4 mm LCP Volar Distal Radius Plate), V plate (2.4 mm LCP Two-Column Volar Distal Radius Plate) and *π* Plate (2.4 mm Volar Rim Distal Radius Plate) (all from Depuy Synthes, West Chester, PA, USA, Ti6Al4V) were built in 3D-matic software. After assembling the internal fixation and fractures, we imported these models into the finite element analysis software (ABAQUS). An axial loading of 100 N was added to the distal end of each model. The displacements of total models and implants, the principal strains and the von Mises stresses in the plates were calculated and compared to capture the biomechanical features of the three plates.

**Results:** The T plate, V plate and *π* plate represented a model displacement of 0.8414 mm, 1.134 mm and 1.936 mm, respectively. The T plate was with the implant displacement of 0.7576 mm, followed by the V plate (0.8802 mm) and the *π* plate (1.545 mm). The T plate had the smallest principal strain of 0.23%, the V plate showed an intermediate level of 0.28%, and the *π* plate had a value of 0.72%. The least peak von Mises stress was observed in the V plate with 263.6MPa, and this value was 435.6 MPa and 1050 MPa in the T plate and *π* plate, respectively.

**Conclusion:** The biomechanical features of three Ti6Al4V volar locking plates in an AO type 23-A3 fracture were described in our analysis. The T plate and the V plate showed similar biomechanical performance while the *π* plate represented worse performance than the other two plates.

## 1 Introduction

Distal radius fractures are the second most common type of fracture in elderly population, accounting for almost 10%–25% of all fractures, and the prevalence increases yearly for all-age individuals (Letsch et al., 2003; Nellans et al., 2012). These fractures usually necessitate surgical interventions to obtain secure fixation.

Open reduction and internal fixation with locking plates is recommended to be the standard procedure for distal radius fractures according to the consensus of the American Academy of Orthopaedic Surgeons (Hammert et al., 2013). However, the selection of plates for internal fixation continues to be a subject of debate, given the variety of plates with different designs and materials available for clinical use. Of these materials, Ti6Al4V is known to provide a lower stress-shielding effect to the bone as compared with the stainless steel due to its low-profile stiffness (Mugnai et al., 2018). Ahirwar et al. developed a femoral fracture model using the finite element method to compare biomechanical performance of Ti6Al4V plates and stainless steel plates and found that Ti6Al4V plates represented a lower deformation and stress (Ahirwar et al., 2021). Though a promising material, polyetheretherketone (PEEK), has been used for plates at present, a 3-year follow-up comparative study showed no difference in clinical outcomes between Ti6Al4V plates and PEEK plates (Berger-Groch et al., 2021). Ti6Al4V is still the most applied material.

In terms of plate designs, the use of double dorsal plates is a traditional technique for the management of distal radius fractures, based on the three-column distal radius and ulna concept to achieve early and secure fixation. This approach facilitates the exposure of posterior displaced fragments and the implantation of internal fixation. However, dorsal plates can be associated with a relatively larger surgical dissection and a higher risk of tendon irritation (Knežević et al., 2017). Currently, volar locking plates are the most common type of internal fixation for distal radius fractures, accounting for 80% of the treatments (Miyashima et al., 2019). This type of internal fixation is preferred by many researchers due to its reduced risk of tendon complications and superior biomechanical stability. A meta-analysis of 38 studies conducted by found that distal radius fractures treated with volar plates exhibited lower complication rates and higher hand function scores compared to dorsal plates (Beyer et al., 2021).

Many volar locking plates are commercially available now, and some researchers have compared their stability and biomechanical features to determine the appropriate selection of plates for different types of distal radius fractures (Koh et al., 2006; Kamei et al., 2010). Reported favorable clinical results in patients with volar rim fractures (AO type 23-B3) using volar plates (Kachooei et al., 2016). Found that volar plates could also provide satisfactory outcomes in cases of dorsally comminuted distal radius fractures, as compared with dorsal plates (Chou et al., 2011). While recent studies have reported positive results with the latest-generation volar plates (Yamamoto et al., 2017; Alter and Ilyas, 2018; Selles et al., 2021), information is lacking on the mechanical attributes of volar plates, particularly with regard to the comparison among different designs. The finite element analysis is a useful tool for evaluating fracture models as it can simulate mechanical responses in a controllable manner and offer reliable data on the biomechanical behaviors of different models. This method is popular for evaluating fracture models (Liu et al., 2020). Used a finite element analysis to investigate the stability of distal radius fractures by volar and dorsal planting (Ghaem-Maghami et al., 2021). The present study aimed to investigate the mechanical performance of three widely-used volar plates with the latest designs using a non-linear finite element analysis.

## 2 Methods

### 2.1 Model construction

**FIGURE 1:**
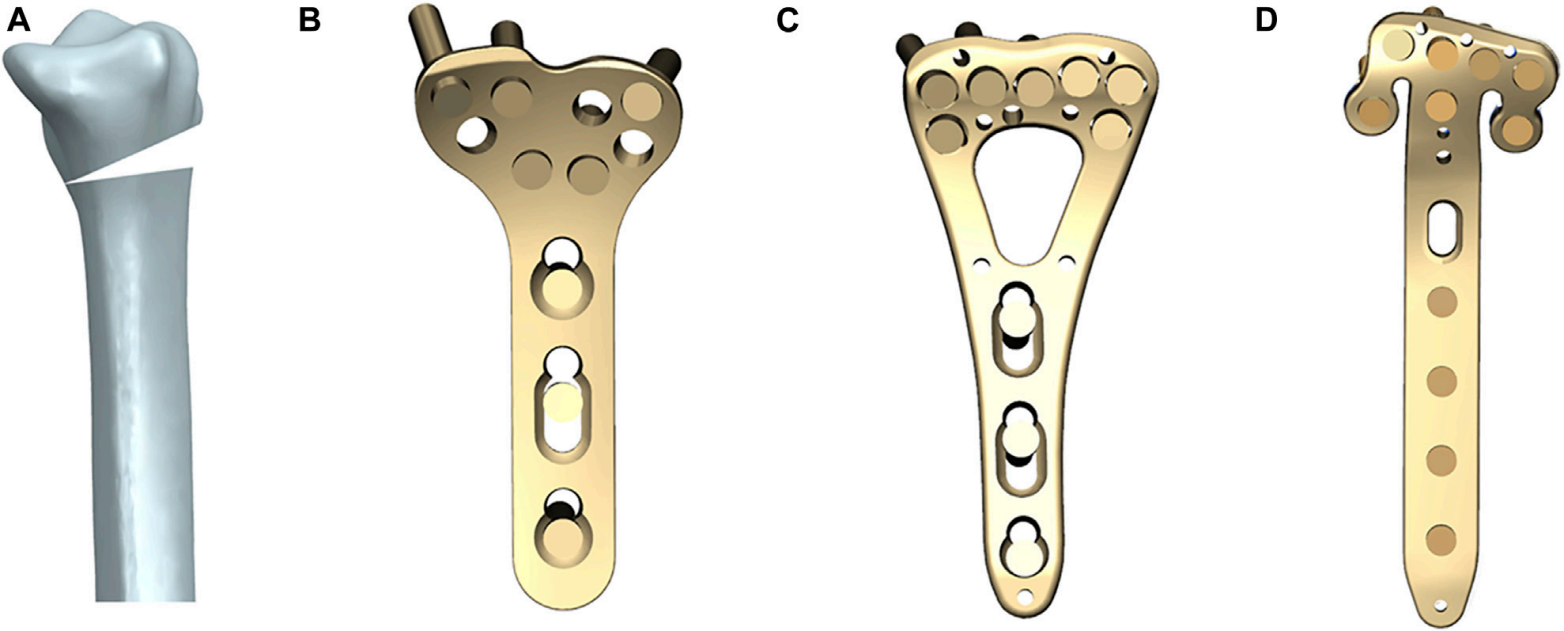
The models of a distal radius fracture and three volar plates (all from Depuy Synthes, West Chester, PA, United States, Ti6Al4V). **(A)** AO type 23-A3 distal radius fracture built by a 10 mm dorsal wedge osteotomy; **(B)** 2.4 mm LCP Volar Distal Radius Plate (T plate); **(C)** 2.4 mm LCP Two-Column Volar Distal Radius Plate (V plate); **(D)** 2.4 mm Volar Rim Distal Radius Plate (π Plate).

In this analysis, three finite element models of extra-articular distal radius fractures (AO type 23-A3) were utilized. The geometric model of the radius was derived from a computed tomography (CT) scan from a 50-year-old female patient who underwent CT angiography for the upper extremities, with ethical approval from our institutional review board (S2020-114-04). The helical CT scan was performed with a slice thickness of 1.0 mm and an interval of 0.8 mm (TOSHIBA Aquilion) and the data was restored as DICOM format and imported into 3D-matic (Materialize, Belgian). The three-dimensional reconstruction of the distal radius was then completed. Subsequently, an extra-articular distal radius fracture was built by a 10 mm dorsal wedge osteotomy (Synek et al., 2021) (Figure 1A). According to the method by (Baumbach et al., 2012), we produced a transverse osteotomy plane at a point 20 mm below the articular surface, and a 10 mm dorsal opening was created.

Geometric three-dimensional models of three volar plates with different designs (T Plate, V Plate and *π* Plate) and screws were reconstructed using the Unigraphics NX 8.5 software (Siemens PLM Software, Co., Ltd, Plano, TX, United States) based on the vendor-provided engineering drawings. The T plate is a 2.4 mm LCP Volar Distal Radius Plate (Figure 1B), the V plate is a 2.4 mm LCP Two-Column Volar Distal Radius Plate (Figure 1C) and the *π* Plate is a 2.4 mm Volar Rim Distal Radius Plate (Figure 1D) (all from Depuy Synthes, West Chester, PA, United States, Ti6Al4V), all of which are anatomically-contoured low-profile implants. The screws were created without threads and assumed to be 20 mm long, and have continuous connections with plates, cortical and cancellous bones, for the purpose of model simplification. The screw insertion was in accordance with the vendor’s recommendations.

We assembled the implants and bones in 3D-matic. The models were exported and then meshed using the HyperMesh 11.0 software (Altair Engineering, Inc., Troy, MI, United States) and imported into the ABAQUS software (Simulia, Suresnes, France).

### 2.2 Assumption and boundary settings

**FIGURE 2:**
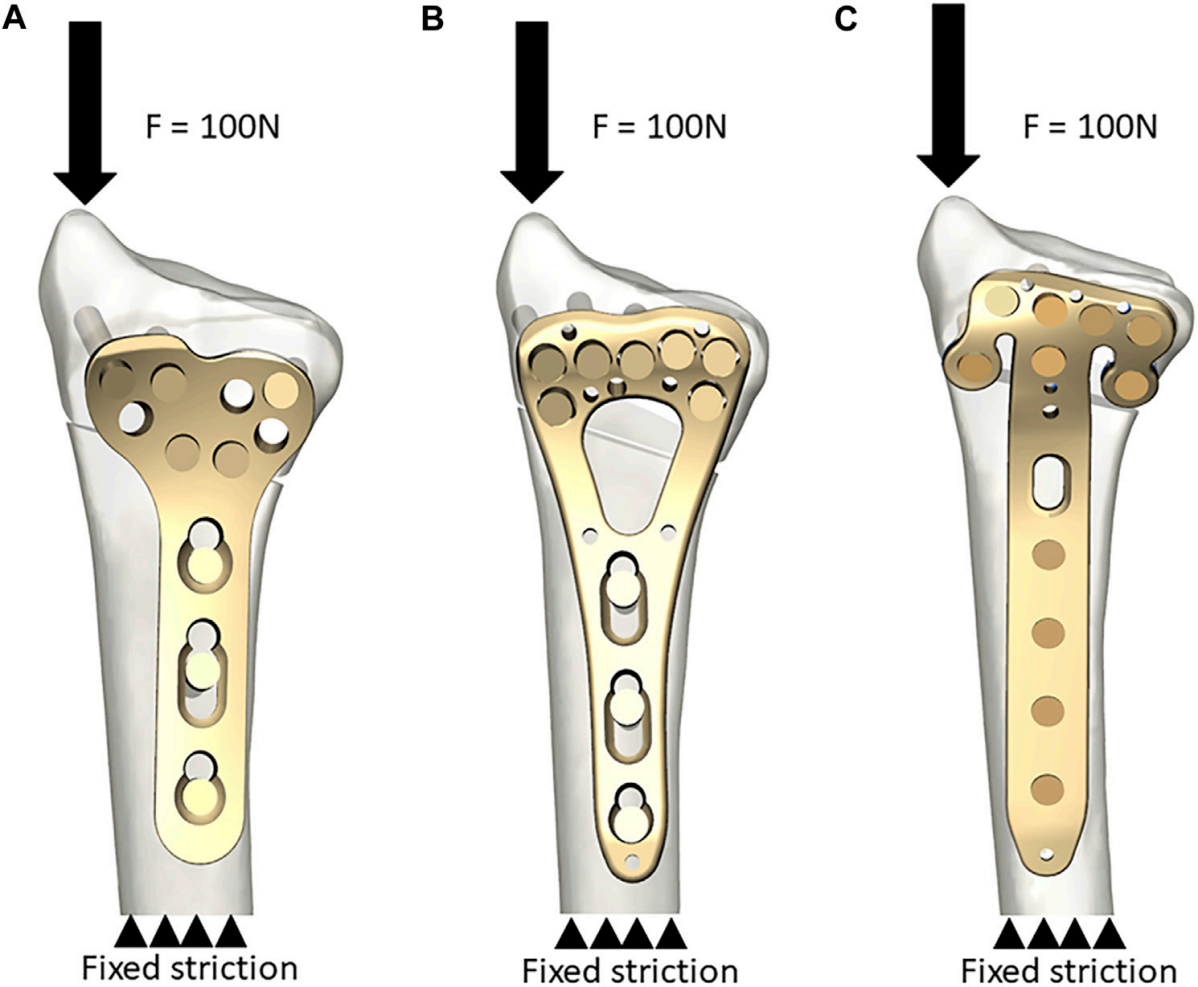
Model construction. Model A was used to clarify the simulation procedure. **(A)** The T plate. An axial load of 100 N was added to the distal end and the proximal end was fixed in all directions; **(B)** The V plate; **(C)** The *π* plate.

Each model was 10 cm long with the proximal end fixed in all directions (Figure 2). Non-linear contact interactions were implementedto mimic the interfacial adaptation between the plates and bones as well as between the osteotomy sites, with a friction coefficient of 0.3 (Liu et al., 2020).

Bonded constraints were employed to connect the screws to the plates and the screws to the bones. The plates were meshed using quadratic tetrahedral elements. The total number of elements and nodes, which varied depending on the type of plate, ranged from 1185403 to 1238464 and from 256012 to 265991, respectively. The material properties were assumed to be isotropic and linear. The elastic modulus of plates and screws was 105 GPa with a Poisson’s ratio of 0.35. The elastic moduli used for the cortical and trabecular bone were 16.7 GPa and 0.155 GPa, respectively. The Poisson’s ratio for cortical bone and trabecular bone was set at 0.26 and 0.3, respectively (Zhang et al., 2018).

### 2.3 Assessment and output parameters

Non-linear computational simulations were conducted using an axial load of 100 N to the distal end to facilitate the identification of reference points (Liu et al., 2020; Synek et al., 2021). The displacements of total models and implants were calculated to reflect the stability of internal fixation systems (Lv et al., 2022). The principal strains and the peak von Mises stresses of plates were also determined and compared to evaluate the biomechanical performance (Wong et al., 2020; Zheng et al., 2022). The values obtained from the model of T plate were used as references, as recommended by Klos et al. (Klos et al., 2010).

## 3 Results

Different biomechanical performance was observed on three models after the axial load simulation (Table 1).

**TABLE 1:** Analysis results after axial loading simulation.

Parameters	T plate	V plate	π plate	V/T (ratio)	π/T (ratio)
Displacement of total models	0.8414 mm	1.134 mm	1.936 mm	1.35	2.30
Displacement of implants	0.7576 mm	0.8802 mm	1.545 mm	1.16	2.04
Principal strain in plates	0.2268%	0.2809%	0.7226%	1.24	3.19
Peak von Mises stress in plates	435.6 MPa	263.6 MPa	1050 MPa	0.61	2.41

**FIGURE 3:**
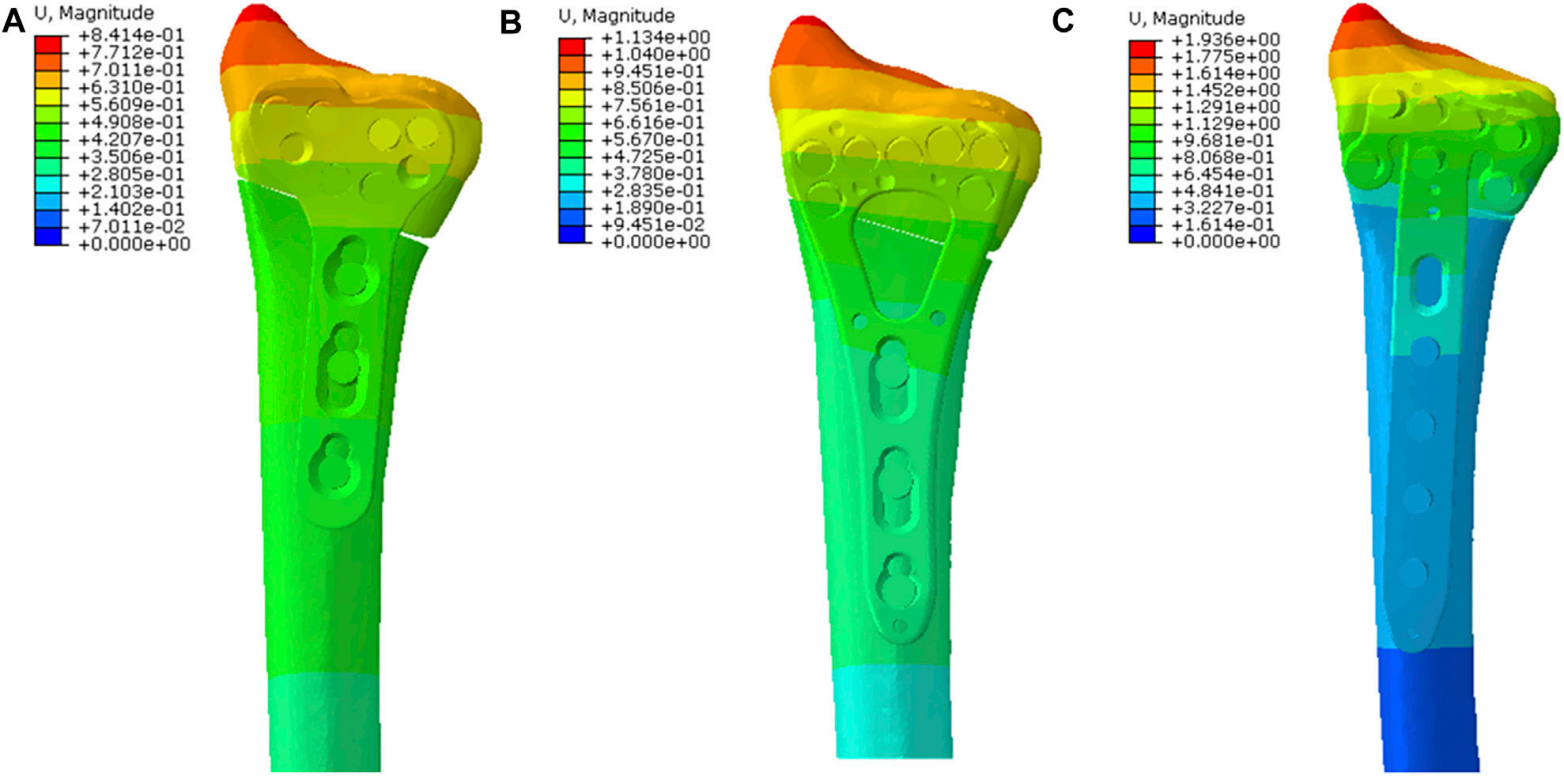
The displacement nephogram of total models and the most obvious displacement was observed at the distal end. **(A)** The nephogram in the T plate; **(B)** The nephogram in the V plate and the total displacement was similar to that in the T plate; **(C)** The nephogram in the *π* plate and the total displacement was higher than those in the other two plates.

**FIGURE 4:**
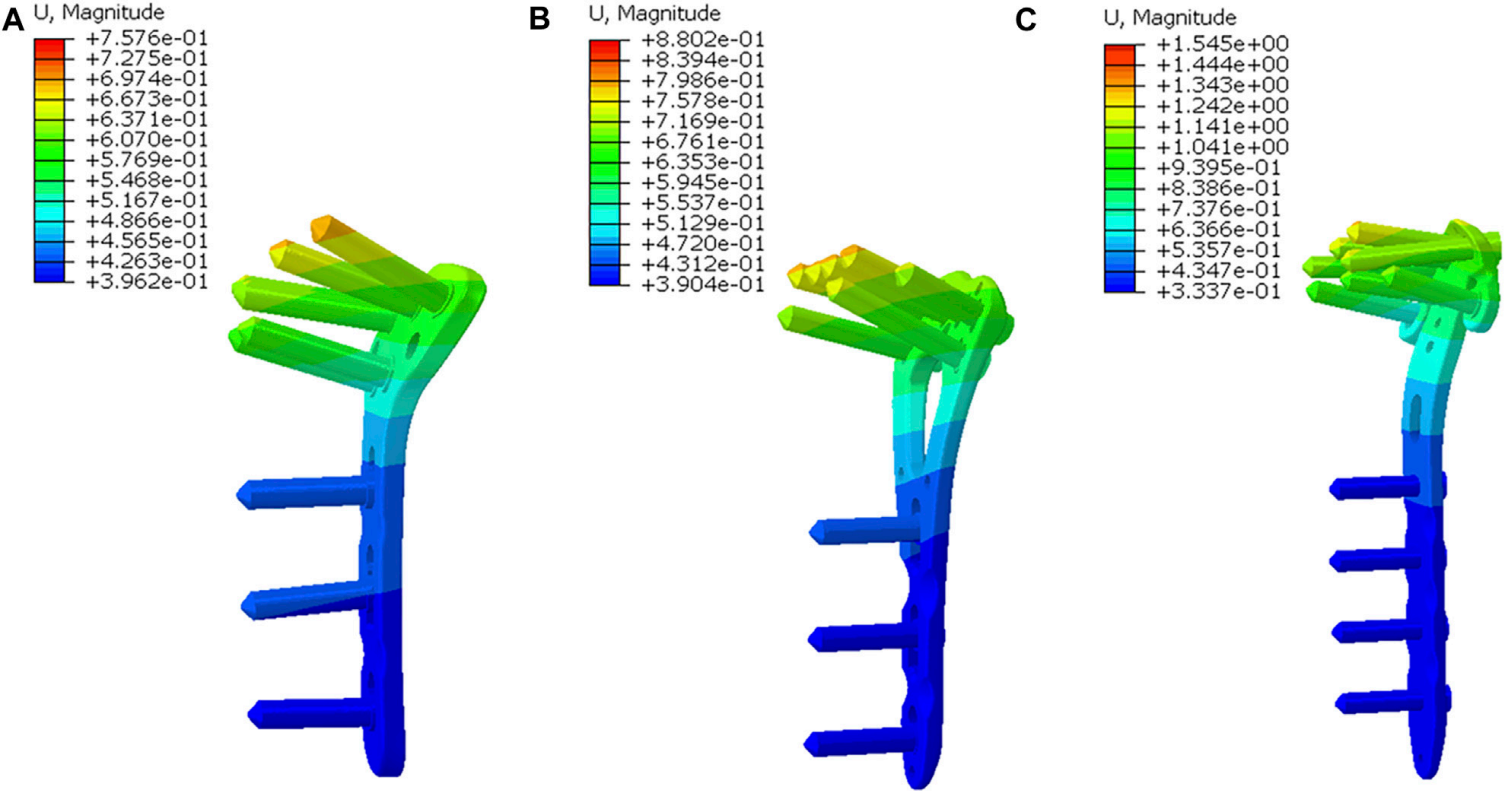
The displacement nephogram of implants. **(A)** The nephogram in the T plate; **(B)** The nephogram in the V plate and the plate displacement was similar to that in the T plate; **(C)** The nephogram in the *π* plate and the plate displacement was higher than those in the other two plates.

The T plate represented the lowest model displacement of 0.8414 mm, while the V plate and the *π* plate displayed 1.4 times and 2.3 times more model displacement than the T plate, respectively (Figure 3). The T plate also had the smallest implant displacement of 0.7576mm, followed by the V plate (0.8802 mm) and the *π* plate (1.545 mm) (Figure 4).

**FIGURE 5:**
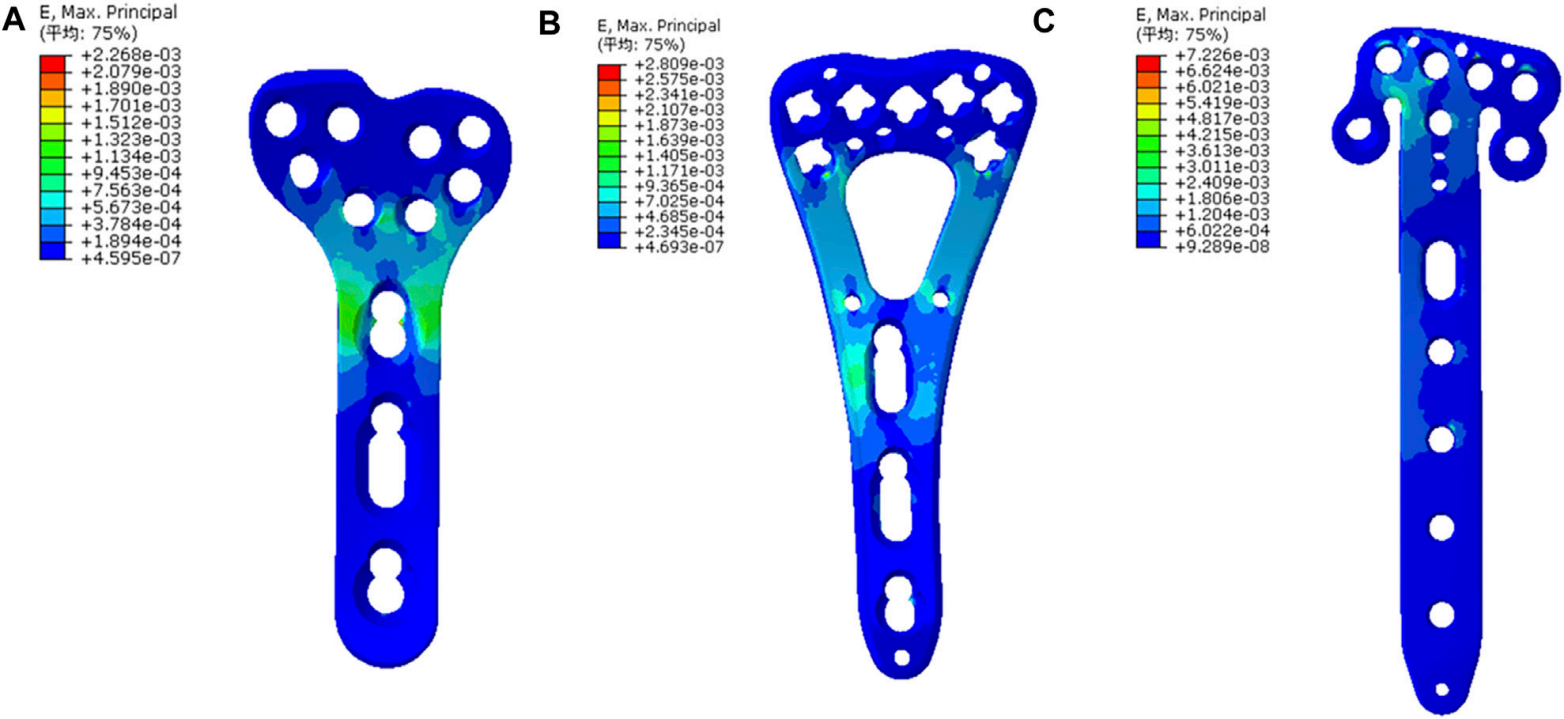
The strain nephogram of implants and the most obvious strain distribution was observed at the junction of plate head and body. **(A)** The strain nephogram in the T plate; **(B)** The nephogram in the V plate and the principal strain was similar to that in the T plate. **(C)** The nephogram in the *π* plate and the principal strain was higher than those in the other two plates.

**FIGURE 6:**
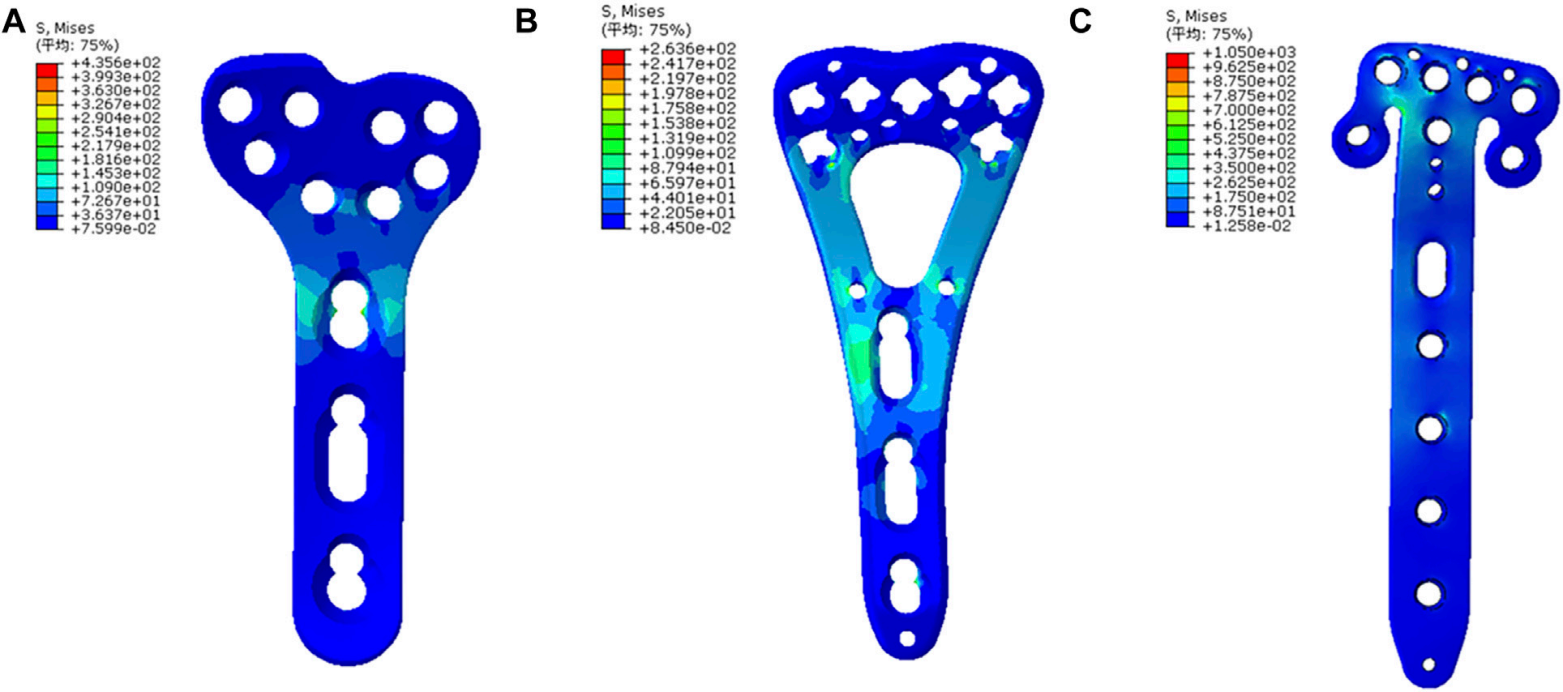
The von Mises stress nephogram of implants and the stresses were mainly concentrated at the junction of plate head and body. **(A)** The stress nephogram in the T plate; **(B)** The nephogram in the V plate and the peak stress was lower than that in the T plate. **(C)** The nephogram in the *π* plate and the peak stress was higher than those in the other two plates.

The principal strains were concentrated at the similar area, the junction of plate head and body, in the three models. The T plate had the smallest strain of 0.23%, with the V plate displaying an intermediate level of 0.28% and the *π* plate showing the highest strain of 0.72% (Figure 5). Peak von Mises stresses of three plates were also concentrated at the junction site. In this regard, the least peak von Mises stress was observed in the V plate with 263.6 MPa, which was 61% of that of the T plate (435.6 MPa). The *π* plate represented a peak von Mises stress of 1050 MPa (Figure 6).

## 4 Discussion

Nearly one-sixth of all fractures in the emergency room are distal radius fractures (Bunch et al., 2016), which can be managed through various modalities including closed reduction, intramedullary fixation, external fixation and open reduction and internal fixation. Among these treatment strategies, open reduction and internal fixation with Ti6Al4V plates has shown faster recovery and improved wrist alignment (Oldrini et al., 2022). Volar locking plates are the most commonly employed type of plates, because of their lower incidences of tendon irritation and superior biomechanical stability over dorsal locking plates. However, to the best of our knowledge, a comprehensive comparison of volar locking plates with different designs has yet to be thoroughly investigated.

Ti6Al4V is one of the most commonly used biomaterials for internal fixation implants. Performed a comparative study regarding the stiffness of three volar plates by Ti6Al4V, stainless steel and PEEK using cadaveric models of distal radius fracture (Mugnai et al., 2018). They found that Ti6Al4V plates were associated with a significantly higher load to failure. Evaluated the postoperative Disabilities of the Arm, Shoulder and Hand (DASH) score for patients with distal radius fractures and found that the patients treated with Ti6Al4V plates showed a higher mean DASH score (15.3) *versus* those treated with PEEK plates (13.2), even though the results were not statistically significant (Perugia et al., 2017).

The present study is the first finite element analysis regarding the comparison among three Ti6Al4V volar plates with latest designs for distal radius fractures. We compared these three plates that were widely utilized in clinical procedures, aiming to investigate which type of plates would have better biomechanical features, as there are few clinical or biomechanical comparative studies in this area. We applied a simple and widely-used test, axial loading test, to simulate the loading pattern of distal radius fractures (Varga et al., 2009). This method can quantify resistance against external stress directly (Cheng et al., 2007). In our analysis, we found that most of results were in favor of the T plate. Both the T plate and the V plate exhibited superior advantages in various dimensions, including model displacements, stresses and strains, when compared with the *π* plate. The values of these parameters in the *π* plate were over 2 times amounts than those in the T plate. The findings suggested that distal radius fractures fixed with a *π* plate might be associated with a higher risk of implant displacement and failure, while a T plate might be a more suitable choice for simple extra-articular distal radius fractures. It should also be noted that, though the T plate appeared to be more stable and solid with lower displacement and strain than the V plate, the V plate showed lower peak von Mises stresses, indicating a lower probability of plates breakage. To our knowledge, among the three types of plates, only one case report of plate breakage was encountered in the T-type plate (volar column plate, The Matrix SmartLock Distal Radius system, Stryker, GmbH & Co. KG, Freiburg, Germany) (Yukata et al., 2009). The plate was broken through the fracture line where the screw holes were unfilled. The authors attributed this to a higher stress concentration in the broken site. Conducted a cadaveric biomechanical analysis of distal radius fractures and proved that the hole at the site of osteotomy is potentially a site of weakness in volar plates (Trease et al., 2005). Our findings, which demonstrated that obvious stresses and strains were distributed at the site of osteotomy, aligned with their results and theories.

The evaluation of displacements and strains indicated a slight superiority the T plate over the V plate. The displacement of V plate was 1.2 times greater than that of T plate. Previous publications mainly focused on the biomechanical/clinical outcomes of a single volar plate. Klos et al. recommended the T plate as a reference due to its good clinical outcomes and acceptable biomechanical features (Klos et al., 2010). The study by Khatri et al. reported that all 23 distal radius fractures following internal fixation with a V plate demonstrated excellent postoperative function with minimal complications (Khatri et al., 2016). Compared the biomechanical features of the V plate with those of the dorsal double plates and those of a juxta-articular distal radius volar plate in cadaveric models (Rausch et al., 2011; Rausch et al., 2013). They found that the V plate had higher initial and final stiffness and less loss of reduction after cyclical testing than the juxta-articular plate, and exhibited biomechanically equivalent stiffness to the dorsal double plates. Abdel-Wahed et al. followed up 96 patients with distal radius fractures for a mean period of 14 months and compared the clinical outcomes of T plates *versus* those of V plates. In their study, the V plates yielded slightly higher postoperative DASH scores over the T plates, while the complication rates and survivorships were comparable (Abdel-Wahed et al., 2022). The study by compared the stiffness of T plates and V plates after cyclical loading in sawbone phantoms of distal radius fractures (Stanbury et al., 2012). The authors discovered that in the extra-articular models, the mean load to failure of T plates (1548 N) was significantly less than that of V plates (2154 N). However, in the intra-articular models, the mean load to failure of T plates (2146 N) was found to be higher than that of V plates (1495 N). Our results of peak von Mises stress were partially consistent with their findings regarding the extra-articular models, as our analysis illustrated that the stress in the V plate (263.6 MPa) was lower than that in the T plate (435.6 MPa) when receiving a 100 N axial loading. This might be associated with the cross-sectional sharps of plates. The V plate had two columns at the junction site while the T plate had only one column.

As for the *π* plate, many studies have demonstrated its superior clinical outcomes, particularly for the comminuted or intra-articular distal radius fractures (Kachooei et al., 2016; Spiteri et al., 2017b; Goorens et al., 2017; Chen et al., 2019; Chua et al., 2022). Reported satisfactory outcomes of *π* plates in 26 patients with complex intra-articular distal radius fractures (Spiteri et al., 2017a). No implant failure was observed in their study. Reviewed the records of 36 patients treated with a *π* plate and found that postoperative Lidstrom wrist scores were deemed as “excellent” in 32 patients (Kara et al., 2016). However, there is a lack of studies comparing the clinical outcomes of *π* plates to other plate types, and biomechanical studies on *π* plates are insufficient. In our analysis, the *π* plate showed inferior outcomes including larger displacements, stresses and strains than the V plate and T plate. This might be attributed to the more distal placement of the *π* plate and the narrower body passing through the osteotomy site, which could lead to a higher stress concentration. To our knowledge, there have been no clinical comparative analyses between the outcomes of *π* plates and those of V plates or T plates in patients with distal radius fractures. Thus, the further clinical investigation is need to identify the appropriate plate selection.

Our research is not without limitations. First, this is a numerical analysis using the finite element method to compare the biomechanical performance of three specific Ti6Al4V plates. The three-dimensional model was reconstructed based on the data from a 50-year-old female. We did not consider the anatomical variations, bone mineral density or other plate designs. The fracture pattern is a simple transverse extra-articular distal radius fracture. These issues can compromise the generalizability of our results in clinical scenarios. Nevertheless, we contend that our numerical estimation could provide a rough description and serve as a supplement for clinical research that is currently lacking. Second, according to the manufacturer (Depuy Synthes), the three plates in our study are intended for the fixation of both intra- and extra-articular fractures. However, some researchers have raised concerns regarding the consistency of the indications for these plates (Soong et al., 2011). Inadequate overlap in indications may also jeopardize the performance of each plate in specific situations. Third, we simplified the models in following aspects: (i) all screws were added to the plate using the assemble function and thus, each screw was placed along the center of each hole; (ii) all screws were modified without threads; (iii) the models did not comprise tendons and thus, the forces transferred onto the bone *via* attached tendons were not taken into account. Fourth, we only performed the axial loading test to simulate the loading pattern after fracture fixation. This method can not reflect the biomechanical properties of plates under loads in other directions, or multiple repetitive loads, which can be investigated by the cycling loading test. Additionally, the torsional loading simulation was not performed, which is important when considering the movement of the forearm in pronation and supination.

## 5 Conclusion

Our analysis compared the biomechanical features of three Ti6Al4V volar locking plates in an AO type 23-A3 fracture, and the results may provide information for surgeons when identifying the optimal plates among different designs for this particular type of fracture. Our findings indicated that the T plate was associated with a smaller model displacement, lower strain and higher stress over the V plate, though their overall performance was comparable. In contrast, the *π* plate appeared to be less effective than the two aforementioned plates in the scenarios of simple extra-articular distal radius fractures.

## Data Availability

The original contributions presented in the study are included in the article/supplementary material, further inquiries can be directed to the corresponding author.

## References

[B1] Abdel-Wahed M. Khater A. A. El-Desouky M. A. (2022). Volar locking plate fixation for distal radius fractures: Did variable-angle plates make difference? Int. Orthop. 46 (9), 2165–2176. 10.1007/s00264-022-05469-z 35690670 PMC9372011

[B2] Ahirwar H. Gupta V. K. Nanda H. S. (2021). Finite element analysis of fixed bone plates over fractured femur model. Comput. Methods Biomech. Biomed. Engin 24 (15), 1742–1751. 10.1080/10255842.2021.1918123 34097536

[B3] Alter T. H. Ilyas A. M. (2018). Complications associated with volar locking plate fixation of distal radial fractures. JBJS Rev. 6 (10), e7. 10.2106/jbjs.Rvw.18.00004 30362969

[B4] Baumbach S. F. Dall'Ara E. Weninger P. Antoni A. Traxler H. Dörr M. (2012). Assessment of a novel biomechanical fracture model for distal radius fractures. BMC Musculoskelet. Disord. 13, 252. 10.1186/1471-2474-13-252 23244634 PMC3557151

[B5] Berger-Groch J. Stodtmeister A. C. Petersen J. P. Hoffmann M. (2021). Palmar plating of distal radius fractures: 3-year follow-up with titanium and PEEK plates give similar outcomes. Acta Orthop. Belg 87 (3), 521–527. 10.52628/87.3.18 34808727

[B6] Beyer J. Wynkoop E. Liu J. Ebraheim N. A. (2021). Interventions for distal radius fractures: A meta-analysis of comparison studies. J. Wrist Surg. 10 (5), 440–457. 10.1055/s-0041-1723793 34631298 PMC8489996

[B7] Bunch P. M. Sheehan S. E. Dyer G. S. Sodickson A. Khurana B. (2016). A biomechanical approach to distal radius fractures for the emergency radiologist. Emerg. Radiol. 23 (2), 175–185. 10.1007/s10140-015-1363-0 26564022

[B8] Chen M. Gittings D. J. Yang S. Liu G. Xia T. (2019). Variable-Angle locking compression plate fixation of distal radius volar rim fractures. Iowa Orthop. J. 39 (2), 55–61. 32577108 PMC7047294

[B9] Cheng H. Y. Lin C. L. Lin Y. H. Chen A. C. (2007). Biomechanical evaluation of the modified double-plating fixation for the distal radius fracture. Clin. Biomech. (Bristol, Avon) 22 (5), 510–517. 10.1016/j.clinbiomech.2006.12.010 17328995

[B10] Chou Y. C. Chen A. C. Chen C. Y. Hsu Y. H. Wu C. C. (2011). Dorsal and volar 2.4-mm titanium locking plate fixation for AO type C3 dorsally comminuted distal radius fractures. J. Hand Surg. Am. 36 (6), 974–981. 10.1016/j.jhsa.2011.02.024 21549526

[B11] Chua W. S. Hassan S. Anoar A. F. (2022). Incidence of flexor tendon injuries in complex intra-articular distal radius fractures fixed with volar rim plate osteosynthesis. Cureus 14 (10), e29852. 10.7759/cureus.29852 36337775 PMC9627411

[B12] Ghaem-Maghami A. Fallah E. Namazi H. Karimi M. T. Hosseini S. I. (2021). The comparison of biomechanical volar and dorsal plating in distal Part Radius fractures; a finite element analysis study. Bull. Emerg. Trauma 9 (1), 9–14. 10.30476/beat.2020.86681 33937420 PMC8062893

[B13] Goorens C. K. Geeurickx S. Wernaers P. Staelens B. Scheerlinck T. Goubau J. (2017). Midterm follow-up of treating volar marginal rim fractures with variable angle lcp volar rim distal radius plates. J. Hand Surg. Asian Pac 22 (2), 184–187. 10.1142/s0218810417500228 28506180

[B14] Hammert W. C. Kramer R. C. Graham B. Keith M. W. (2013). AAOS appropriate use criteria: Treatment of distal radius fractures. J. Am. Acad. Orthop. Surg. 21 (8), 506–509. 10.5435/jaaos-21-08-506 23908257

[B15] Kachooei A. R. Tarabochia M. Jupiter J. B. (2016). Distal radius volar rim fracture fixation using DePuy-synthes volar rim plate. J. Wrist Surg. 5 (1), 002–008. 10.1055/s-0035-1570740 PMC4742267 26855829

[B16] Kamei S. Osada D. Tamai K. Kato N. Takai M. Kameda M. (2010). Stability of volar locking plate systems for AO type C3 fractures of the distal radius: Biomechanical study in a cadaveric model. J. Orthop. Sci. 15 (3), 357–364. 10.1007/s00776-010-1466-0 20559804

[B17] Kara A. Celik H. Oc Y. Uzun M. Erdil M. Tetik C. (2016). Flexor tendon complications in comminuted distal radius fractures treated with anatomic volar rim locking plates. Acta Orthop. Traumatol. Turc 50 (6), 665–669. 10.1016/j.aott.2016.04.001 27836497 PMC6197461

[B18] Khatri K. Sharma V. Farooque K. Tiwari V. (2016). Surgical treatment of unstable distal radius fractures with a volar variable-angle locking plate: Clinical and radiological outcomes. Arch. Trauma Res. 5 (2), e25174. 10.5812/atr.25174 27679785 PMC5035514

[B19] Klos K. Rausch S. Löffler M. Fröber R. Hofmeier K. Lenz M. (2010). A biomechanical comparison of a biodegradable volar locked plate with two titanium volar locked plates in a distal radius fracture model. J. Trauma 68 (4), 984–991. 10.1097/TA.0b013e3181b28962 20016391

[B20] Knežević J. Kodvanj J. Čukelj F. Pamuković F. Pavić A. (2017). A biomechanical comparison of four fixed-angle dorsal plates in a finite element model of dorsally-unstable radius fracture. Injury 48 (5), S41–s46. 10.1016/s0020-1383(17)30738-6 29122121

[B21] Koh S. Morris R. P. Patterson R. M. Kearney J. P. Buford W. L. Jr. Viegas S. F. (2006). Volar fixation for dorsally angulated extra-articular fractures of the distal radius: A biomechanical study. J. Hand Surg. Am. 31 (5), 771–779. 10.1016/j.jhsa.2006.02.015 16713841

[B22] Letsch R. Infanger M. Schmidt J. Kock H. J. (2003). Surgical treatment of fractures of the distal radius with plates: A comparison of palmar and dorsal plate position. Arch. Orthop. Trauma Surg. 123 (7), 333–339. 10.1007/s00402-003-0538-4 12819989

[B23] Liu H. C. Jiang J. S. Lin C. L. (2020). Biomechanical investigation of a novel hybrid dorsal double plating for distal radius fractures by integrating topology optimization and finite element analysis. Injury 51 (6), 1271–1280. 10.1016/j.injury.2020.03.011 32268963

[B24] Lv M. L. Ni M. Sun W. Wong D. W. Zhou S. Jia Y. (2022). Biomechanical analysis of a novel double-point fixation method for displaced intra-articular calcaneal fractures. Front. Bioeng. Biotechnol. 10, 791554. 10.3389/fbioe.2022.791554 35356772 PMC8959616

[B25] Miyashima Y. Kaneshiro Y. Yano K. Teraura H. Sakanaka H. Uemura T. (2019). Size and stabilization of the dorsoulnar fragment in AO C3-type distal radius fractures. Injury 50 (11), 2004–2008. 10.1016/j.injury.2019.08.003 31427036

[B26] Mugnai R. Tarallo L. Capra F. Catani F. (2018). Biomechanical comparison between stainless steel, titanium and carbon-fiber reinforced polyetheretherketone volar locking plates for distal radius fractures. Orthop. Traumatol. Surg. Res. 104 (6), 877–882. 10.1016/j.otsr.2018.05.002 29807189

[B27] Nellans K. W. Kowalski E. Chung K. C. (2012). The epidemiology of distal radius fractures. Hand Clin. 28 (2), 113–125. 10.1016/j.hcl.2012.02.001 22554654 PMC3345129

[B28] Oldrini L. M. Feltri P. Albanese J. Lucchina S. Filardo G. Candrian C. (2022). Volar locking plate vs cast immobilization for distal radius fractures: A systematic review and meta-analysis. EFORT Open Rev. 7 (9), 644–652. 10.1530/eor-22-0022 36125012 PMC9624483

[B29] Perugia D. Guzzini M. Mazza D. Iorio C. Civitenga C. Ferretti A. (2017). Comparison between Carbon-Peek volar locking plates and titanium volar locking plates in the treatment of distal radius fractures. Injury 48 (3), S24–s29. 10.1016/s0020-1383(17)30653-8 29025605

[B30] Rausch S. Schlonski O. Klos K. Gras F. Gueorguiev B. Hofmann G. O. (2013). Volar versus dorsal latest-generation variable-angle locking plates for the fixation of AO type 23C 2.1 distal radius fractures: A biomechanical study in cadavers. Injury 44 (4), 523–526. 10.1016/j.injury.2012.08.048 23000052

[B31] Rausch S. Klos K. Stephan H. Hoffmeier K. Gras F. Windolf M. (2011). Evaluation of a polyaxial angle-stable volar plate in a distal radius C-fracture model--a biomechanical study. Injury 42 (11), 1248–1252. 10.1016/j.injury.2010.12.005 21329924

[B32] Selles C. A. Mulders M. A. M. Winkelhagen J. van Eerten P. V. Goslings J. C. Schep N. W. L. (2021). Volar Plate fixation versus cast immobilization in acceptably reduced intra-articular distal radial fractures: A randomized controlled trial. J. Bone Jt. Surg. Am. 103 (21), 1963–1969. 10.2106/jbjs.20.01344 34314402

[B33] Soong M. Earp B. E. Bishop G. Leung A. Blazar P. (2011). Volar locking plate implant prominence and flexor tendon rupture. J. Bone Jt. Surg. Am. 93 (4), 328–335. 10.2106/jbjs.J.00193 21239658

[B34] Spiteri M. Ng W. Matthews J. Power D. (2017a). Functional outcome of fixation of complex intra-articular distal radius fractures with a variable-angle distal radius volar rim plate. J. Hand Microsurg 9 (1), 011–016. 10.1055/s-0037-1601325 PMC5403733 28442856

[B35] Spiteri M. Roberts D. Ng W. Matthews J. Power D. (2017b). Distal radius volar rim plate: Technical and radiographic considerations. World J. Orthop. 8 (7), 567–573. 10.5312/wjo.v8.i7.567 28808628 PMC5534406

[B36] Stanbury S. J. Salo A. Elfar J. C. (2012). Biomechanical analysis of a volar variable-angle locking plate: The effect of capturing a distal radial styloid fragment. J. Hand Surg. Am. 37 (12), 2488–2494. 10.1016/j.jhsa.2012.09.009 23174062

[B37] Synek A. Baumbach S. F. Pahr D. H. (2021). Towards optimization of volar plate fixations of distal radius fractures: Using finite element analyses to reduce the number of screws. Clin. Biomech. (Bristol, Avon) 82, 105272. 10.1016/j.clinbiomech.2021.105272 33493739

[B38] Trease C. McIff T. Toby E. B. (2005). Locking versus nonlocking T-plates for dorsal and volar fixation of dorsally comminuted distal radius fractures: A biomechanical study. J. Hand Surg. Am. 30 (4), 756–763. 10.1016/j.jhsa.2005.04.017 16039369

[B39] Varga P. Baumbach S. Pahr D. Zysset P. K. (2009). Validation of an anatomy specific finite element model of Colles' fracture. J. Biomech. 42 (11), 1726–1731. 10.1016/j.jbiomech.2009.04.017 19467661

[B40] Wong C. E. Hu H. T. Hsieh M. P. Huang K. Y. (2020). Optimization of three-level cervical hybrid surgery to prevent adjacent segment disease: A finite element study. Front. Bioeng. Biotechnol. 8, 154. 10.3389/fbioe.2020.00154 32195235 PMC7064443

[B41] Yamamoto M. Fujihara Y. Fujihara N. Hirata H. (2017). A systematic review of volar locking plate removal after distal radius fracture. Injury 48 (12), 2650–2656. 10.1016/j.injury.2017.10.010 29031822

[B42] Yukata K. Doi K. Hattori Y. Sakamoto S. (2009). Early breakage of a titanium volar locking plate for fixation of a distal radius fracture: Case report. J. Hand Surg. Am. 34 (5), 907–909. 10.1016/j.jhsa.2009.01.004 19410996

[B43] Zhang W. Li J. Zhang H. Wang M. Li L. Zhou J. (2018). Biomechanical assessment of single liss versus double-plate osteosynthesis in the AO type 33-C2 fractures: A finite element analysis. Injury 49 (12), 2142–2146. 10.1016/j.injury.2018.10.011 30322705

[B44] Zheng L. Wang C. Hu M. Apicella A. Wang L. Zhang M. (2022). An innovative additively manufactured implant for mandibular injuries: Design and preparation processes based on simulation model. Front. Bioeng. Biotechnol. 10, 1065971. 10.3389/fbioe.2022.1065971 36507282 PMC9729797

